# A Brief, Daily, Online Mental Health and Well-being Intervention for University Staff During the COVID-19 Pandemic: Program Description and Outcomes Using a Mixed Methods Design

**DOI:** 10.2196/35776

**Published:** 2022-02-25

**Authors:** Alexandra Parker, Sarah Dash, Matthew Bourke, Rhiannon Patten, Melinda Craike, Peter Baldwin, Warwick Hosking, Itamar Levinger, Vasso Apostolopoulos, Maximilian de Courten, Jenny Sharples, Monika Naslund, Vasileios Stavropoulos, Mary Woessner, Christopher Sonn, Caroline Stansen, Michaela Pascoe

**Affiliations:** 1 Institute for Health and Sport Victoria University Melbourne Australia; 2 Orygen Centre for Youth Mental Health University of Melbourne Melbourne Australia; 3 Mitchell Institute Victoria University Melbourne Australia; 4 Black Dog Institute Sydney Australia; 5 Australian Institute for Musculoskeletal Science Department of Medicine, Western Health Melbourne Medical School, University of Melbourne Melbourne Australia; 6 Peter MacCallum Cancer Centre Melbourne Australia

**Keywords:** workplace mental health, well-being, mental health promotion, online intervention, telehealth, COVID-19 pandemic, COVID-19, pandemic, health promotion

## Abstract

**Background:**

The unprecedented changes and isolation measures to contain COVID-19 have had multiple psychological and social impacts, with implications for professional and personal functioning. Evidence-informed interventions that can be rapidly implemented under pandemic conditions to support mental health during such times are urgently needed.

**Objective:**

The aim of this study was to determine the acceptability and preliminary outcomes of a daily online mental health promotion program for tertiary education staff during the COVID-19 pandemic.

**Methods:**

The “Victoria University (VU) Elevenses” program was delivered as an uncontrolled intervention at Victoria University (VU) in the western metropolitan region of Melbourne, Australia. In April 2020, an email invitation was sent to all academic and professional staff inviting them to: (1) participate in the program and (2) opt-in to the research component. The “VU Elevenses” program provided 10-15–minute microinterventions comprising lifestyle and well-being strategies to promote mental health via an online meeting platform at 11 AM each weekday. A mixed methods approach was used to evaluate the program, combining structured questionnaires with semistructured interviews to investigate the experiences of staff who participated in the program.

**Results:**

Between 16 and 90 participants provided weekly program feedback. A total of 106 university staff opted into the longitudinal research component and 10 staff participated in the interviews. Participants reported high levels of satisfaction with sessions and perceived benefits for mental health. Approximately one quarter of participants reported moderate to severe symptoms of depression, anxiety, and stress at baseline, with significant reductions in these symptoms in the first 7 weeks of the program, corresponding with easing in mandatory isolation (“lockdown”) restrictions. Symptoms of depression, anxiety, and stress all increased when lockdown measures were reintroduced, but not to the same levels as found during the initial lockdown period. Overall changes in depression and anxiety from baseline to the end of the program were explained by changes in COVID-19–related distress, whereas changes in self-compassion explained changes in stress.

**Conclusions:**

We show that it is feasible and acceptable to develop and deliver a program of brief interventions in a timely manner, using a simple and accessible online platform. Although participation in the program was initially associated with reduced symptoms of depression, anxiety, and stress, participants’ mental health worsened with the reintroduction of a “lockdown” period. However, as symptoms of depression, anxiety, and stress did not return to levels observed at the start of the VU Elevenses program, participation in the uncontrolled intervention may have offered a protective benefit against the impact of the second significant lockdown period.

## Introduction

The Australian national response to the global pandemic of COVID-19 has been consistent with international approaches recommending or mandating physical distancing and self-isolation. Psychological and social risks associated with this period of rapid change and uncertainty included the inability to access workplaces; educational institutions; and social, cultural, and sporting events. Psychological stressors include concerns about income, job insecurity, and changes to work practices (eg, remote working, reduced hours, or loss of job), with nearly one third of Australians reporting worsening household finances due to the COVID-19 pandemic during 2020 [1]. Additional health-related anxiety for self and loved ones, the complexity of caring responsibilities (including remote learning/schooling from home), potentially unsafe home environments, and the impacts of social isolation [2] contributed to significant increases in rates of psychological distress compared with Australian national data from 2017 to 2018 [1,3]. Findings from a survey from the beginning of the pandemic of almost 1500 Australian adults indicated that more than 1 in 4 respondents reported depression scores that were moderately to extremely severe, and approximately 1 in 5 had moderately to extremely severe stress during the period of peak COVID-19 self-isolation and physical distancing requirements [4].

Structure and consistency in everyday life are important in preserving mental well-being during enforced periods of self-isolation and uncertainty [5]. Using helpful coping strategies, maintaining daily structure, and staying connected were consistently endorsed by national (eg, Beyond Blue [6]) and international (eg, [5]) health organizations as critical to maintaining mental well-being during the COVID-19 crisis. Further, the delivery of psychological interventions and interactions via online meeting platforms [7], and brief, targeted interventions for well-being have been shown to be effective in promoting mental health in clinical and nonclinical adult populations [8-10]. To promote and maintain physical and mental well-being for staff of a tertiary education institution during the COVID-19 pandemic, we delivered an evidence-informed, timely, accessible, responsive online intervention comprising brief, daily (weekday) microinterventions and strategies targeting six essential lifestyle areas for well-being (healthy eating, physical activity, reducing alcohol intake, improving sleep, healthy relationships and social connection, and stress management) [11]. In deference to the warmth, comfort, and connection shared over a cup of tea and sticky bun (ie, morning tea referred to as “elevenses”) between Paddington Bear and his dear friend, Mr Gruber [12], our program was titled the “Victoria University (VU) Elevenses.” The aim of this study was to determine the acceptability of the program; assess changes in stress, depressive, and anxiety symptoms in program participants over time; and determine mediators of change in symptoms of stress, depression, and anxiety from baseline to the endpoint of the program.

## Methods

### Setting

The study was conducted at Victoria University (VU) in the western metropolitan region of Melbourne, Australia. VU has eight Victorian campuses, with 1994 academic (teaching and research) and professional staff, 988 sessional staff (academic and polytechnic teachers), and 598 casual professional staff. Among the total VU staff, 59.64% (2135/3580) identify as female.

### Participants

All staff received an email invitation to participate in the online program and to opt-in to the research component. All staff were eligible to participate in the program, with no exclusion criteria or minimum attendance requirements. Using the general university mailing list, invitations to join the daily online sessions were issued over the first 2 weeks of the program, with staff asked to opt-in via email to receive ongoing meeting invitations. At the end of the program, all those who opted in to meeting invitations (17.93%, 642/3580) were invited by email to participate in the semistructured interviews.

### Ethics Approval

All procedures involving human participants were approved by the Victoria University Human Research Ethics Committee (HRE20-054) and complied with the ethical standards of this institutional committee.

### Research Design

This study followed a mixed methods design. A repeated-measures within-subjects design was used to collect data on mental health outcomes and potential mediators of change from baseline to 32 weeks after program commencement. Initially, it was planned that follow-up assessments would occur every 3 weeks. However, given that the program ran longer than anticipated due to Melbourne’s extended lockdown period, the repeated-measures time points became longer as the program progressed to reduce participant burden. Additionally, brief weekly questionnaires were used to determine the acceptability of the program over the first 13 weeks of the program. At the end of the program, we also conducted in-depth semistructured interviews in a small sample of participants to explore their experiences with VU Elevenses.

### Intervention Program

The “VU Elevenses” program focused on mental health promotion, including universal, selected, and indicated prevention strategies [13]. Participants were provided information on how to access mental health treatment from existing internal (ie, Employee Assistance Program) and external (ie, Beyond Blue, headspace, Black Dog Institute, Lifeline, Relationships Australia, Family Relationships Advice Line) sources, as the “VU Elevenses” program was not designed to be a treatment for mental health conditions. The program was aligned with the VU Employee Wellbeing Policy [14].

The “VU Elevenses” program comprised brief (10-15 minutes), evidence-informed microinterventions and strategies to promote physical and mental well-being, utilizing an online meeting platform at 11 AM each weekday. The intervention had three main phases: (1) managing immediate concerns and stressors, (2) adjusting to working and studying remotely, and (3) preparing to return to work and study on campus. The design and implementation of the program were guided by the principles of inclusivity and accessibility, responsiveness, consistency, and connectedness. The sessions were live and unpolished to maintain authenticity and connection, and to allow for the rapid sharing of evidence-based clinical content for mental health and well-being support.

A collective of VU practitioners and researchers rotated and delivered interventions relevant to one of six identified lifestyle areas (healthy eating, physical activity, reducing alcohol intake, improving sleep, healthy relationships and social connection, and stress management) [15]. Each microintervention aimed to promote skill-building through simple mindfulness strategies, deep breathing exercises, relaxation exercises, time-management and routine-setting strategies, self-compassion strategies, physical activity guidance, sleep tips, nutrition advice, and fun activities for community connection (eg, quizzes and group singing sessions; see Table 1).

Members of the program team planned the following week of microintervention content, resources, and presenters. Resources for further information (ie, fact sheets, websites, apps, YouTube clips, online interventions) and links to recordings were provided to participants for each daily session via VU’s intranet. The program was evaluated for 32 weeks, from April 2020 to November 2020, with sessions each weekday for the first 24 weeks, followed by three times per week for the final 8 weeks of the program.

**Table 1:** Session content overview for the first three weeks of the “Victoria University (VU) Elevenses” program.

Day^a^	Lifestyle intervention theme	Content	Presenter	Resources shared
1	Stress management	Introduction to "VU Elevenses," psychoeducation on sympathetic and parasympathetic nervous systems, deep breathing exercise (box breathing)	Clinical psychologist/research academic	Links to breathing apps, box breathing jpeg
2	Stress management	Simple mindfulness strategies (Notice 5 Things and Leaves on a Stream)	Clinical psychologist/research academic	Links to mindfulness and meditation apps
3	Relationships	Managing roles and responsibilities	Clinical psychologist/teaching and research academic	Links to resources for maintaining mental health and relationships when working from home (ie, Lifeline and Relationships Australia fact sheets)
4	Physical activity	Stretching exercises for shoulder, neck, and back	Accredited exercise physiologist/research academic	Links to web pages of accredited organizations for home-based stretching exercises
5	Relationships (community connection)	Online quiz (1908s music)	Clinical psychologist/research academic	None
6	Relationships	Schooling from home	Clinical and community psychologist/teaching academic	Links to popular media articles with expert opinions on challenges of remote education
7	Stress management	Resetting workload expectations	Clinical psychologist/teaching and research academic	Links to popular media articles with expert opinions on academic expectations during COVID-19
8	Stress management	Self-compassion meditation	Probationary psychologist/teaching and research academic	Links to website for additional self-compassion resources
9	Physical activity	Exercise “snacking”: 5 minutes of light aerobic activities	Accredited exercise physiologist/research academic	Link to YouTube clip of 5-minute cardio work out
10	Relationships (community connection)	Online quiz (general knowledge)	Clinical psychologist/research academic	None
11	Stress management	Progressive muscle relaxation	Clinical psychologist/research academic	Link to online audio clip of recorded progressive muscle relaxation
12	Sleep	Guidelines and tips to improve sleep quality	Teaching and research academic	Links to sleep guidelines and national Sleep Health Foundation
13	Stress management	Managing time and work commitments during change	Clinical psychologist/research academic	Links to sprint technique (“Pomodoro”) and popular media article on time management
14	Physical activity	Exercise “snacking”: 10-15 minutes of resistance activities	Accredited exercise physiologist/research academic	Links to web pages of accredited organizations for home-based stretching exercises and YouTube home workout clip
15	Relationships (community connection)	Introduction to Indigenous mindfulness practices and connection to country	Indigenous Community Liaison Officer and licensed Wayapa practitioner	Links to Wayapa and YouTube clip from Victorian Aboriginal Heritage Council

^a^Days 16-20 included healthy eating (nutrition and mental health), “desk yoga,” pleasant activity scheduling, exercise “snacking” (light aerobic activities), and a music sing-along.

### Outcomes

#### Program Acceptability

All participants were provided with a link to a brief survey at the end of each week for the first 13 weeks of the intervention. Participants were asked to provide weekly feedback on the acceptability of the intervention by answering the following questions on a scale from 1 (strongly disagree) to 7 (strongly agree): (1) “I am generally satisfied with the sessions this week,” (2) “I found the content helpful for my mental health and well-being,” and (3) “I felt more connected with my colleagues.” To measure engagement, each respondent was asked to report the number of live sessions they attended that week. Attendance at the daily sessions was assessed at week 1, week 16, and week 32 using the online meeting platform’s participant logs.

#### Participant Experiences

One-on-one semistructured interviews were conducted with a sample (n=10) of VU staff members who attended at least one VU Elevenses session, as previous research shows that data saturation for the most part occurs between 6 and 12 interviews [16]. Participants were prompted about their level of engagement, their overall perceptions of the program, and how useful they found the program. The full interview schedule can be seen in Multimedia Appendix 1.

#### Mental Health, Well-being, and Health Behaviors

The mental health of participants was measured using the Short Form Depression, Anxiety and Stress Scale (DASS-21) [17]. The Pittsburgh Sleep Quality Index [18] was used to measure sleep quality; the WHO Alcohol, Smoking & Substance Involvement Screening Test [19] was used to measure alcohol intake; the Coping Scale [20] and Self-Compassion Short Form (SCS-SF) [21] were used to measure stress management; the Multidimensional Scale of Perceived Social Support (MSPSS) [22] and Social Connectedness and the Social Assurance Scales (SCS) [23] were used to measure healthy relationships and social connection; the Starting the Conversation Diet Instrument [24] was used to measure diet quality; and the Active Australia Survey was used to measure current leisure-time physical activity [25]. Additionally, COVID-19–specific distress was measured using the Coronavirus Anxiety Scale [26].

### Analysis of Acceptability and Participants’ Experiences

To determine acceptability of the program, percentages of participants who agreed or strongly agreed they were satisfied with the content of the session, felt the content helped with their mental health and well-being, and thought the program made them feel more connected with their colleagues during the first 13 weeks of the program were calculated. Additionally, the average number of live daily sessions attended by respondents in a week was calculated.

The semistructured interviews were digitally recorded and then transcribed verbatim. Transcripts were read and reread while listening to the recordings to ensure accuracy. Braun and Clarke’s [27] thematic analysis guide was used to examine the personal experiences and meanings of the program. Data were organized in a meaningful and systematic way, with coding used to reduce data into smaller chunks of meaning. Codes were examined and organized into broader themes and were reviewed and modified. The final themes were defined and named.

### Analyses of Mental Health Outcomes

Quantitative data analysis was performed using SPSS (v.25) and the R package. Trajectories of change in symptoms of depression, anxiety, and stress were estimated using multilevel growth curve models. First, an unconditional model with a random intercept was run for each outcome (null model). Subsequently, a series of models were run with a fixed linear effect of time (Model A), quadratic effect of time (Model B), and cubic effect of time (Model C) added. Because the time between follow-ups differed, time was entered as the number of weeks from baseline centered on the date of baseline collection. All models were fitted with a random intercept. Additionally, models were run with random slopes. However, the inclusion of random slopes did not significantly improve the fit of any model; thus, the results are reported for models with a random intercept only. The likelihood ratio test was used to compare model fits and determine which model fit the data best. Before analysis, models were checked for normality. The results indicated that depression and anxiety were not normally distributed, and therefore these outcomes were log-transformed before analysis. Because logs of zeroes cannot be computed, 1 was added to all scores of depression and anxiety before transformations were made.

To determine mediators of changes in depression symptoms from baseline to the end of the program, 1-1-1 multilevel multiple mediation models were estimated using the MLmed macro tool [28]. Models were estimated with random intercepts, fixed slopes, and uncorrelated residuals. The significance of indirect effects was estimated via Monte Carlo 95% CIs based on 10,000 resamples. Variables for each of the six identified lifestyle areas (healthy eating, physical activity, reducing alcohol intake, improving sleep, healthy relationships and social connection, and stress management) were tested as possible mediators. Additionally, to account for potential changes due to fluctuating responses to COVID-19, COVID-19–related distress was also tested as a potential mediator. To keep models parsimonious, the three variables with the strongest within-person correlations [29] with each of the outcomes were included in a single multiple mediation model. Within-person correlations between variables are provided in Multimedia Appendix 2.

### Missing Data

There were large amounts of missing data. Only six participants provided data at the fourth follow-up, and therefore data from this time point were excluded from analyses. Of the remaining five assessments, 43 participants only completed the baseline assessment, 18 participants completed two assessments, 8 participants completed three assessments, 15 participants completed four assessments, and 20 participants completed all five assessments. Data were available for 106 participants at baseline, 51 at the first follow-up, 42 at the second follow-up, 36 at the third follow-up, and 31 at the fifth follow-up. One-way analysis of variance and Kruskal–Wallis tests were used to determine if there was a significant difference in study variables at baseline between participants with complete data and those missing data at follow-up. Missing value analysis indicated no significant differences in symptoms of depression, anxiety, or stress, or in demographics between participants with different amounts of missing data, indicating that the data were missing completely at random. Missing data for symptoms of depression, anxiety, and stress were handled using the full information maximum-likelihood estimation method. This has been shown to reduce parameter errors compared to ad hoc approaches to handling missing data, and provides appropriate standard errors of estimations, even with substantial amounts of missing data and small sample sizes [30,31].

For the mediation analysis, 31 participants completed both the baseline and follow-up assessments. Results indicated that participants who completed both baseline and follow-up assessments reported significantly less COVID-19–related distress at baseline than participants who did not complete the follow-up assessment, indicating that the data were not missing completely at random. Because multilevel models cannot handle missing data for independent variables using maximum likelihood, missing values for mediator variables were imputed using multiple imputation. A total of 10 imputed data sets were created using Markov Chain Monte Carlo with predictive mean matching. Imputation models included all mediators and outcomes as predictors of missing values. Results from the multiple imputed data sets were combined using Rubin’s Rule [32].

## Results

### Program Acceptability

Attendance at the daily sessions ranged from 174 to 240 participants in the first week of the program, 63-84 at week 16, and 21-24 in the final week of the program. The number of responses to weekly feedback ranged from 16 to 90 participants. The average weekly attendance for participants who responded to the weekly feedback questionnaires ranged between 2.38 and 3.22 sessions. Participants reported high levels of satisfaction with the sessions during the first 13 weeks of the study (81.0% and 97.7% of weekly respondents agreeing or strongly agreeing that they were satisfied with the sessions they attended that week), and found the sessions they attended helpful for their mental health and well-being (80.9% and 94.2% of weekly respondents agreeing or strongly agreeing). Increased connection with colleagues was moderately endorsed (46.1% and 94.1% of weekly respondents agreed or strongly agreed).

### Program Experiences

#### Overview of Themes

**Figure 1:**
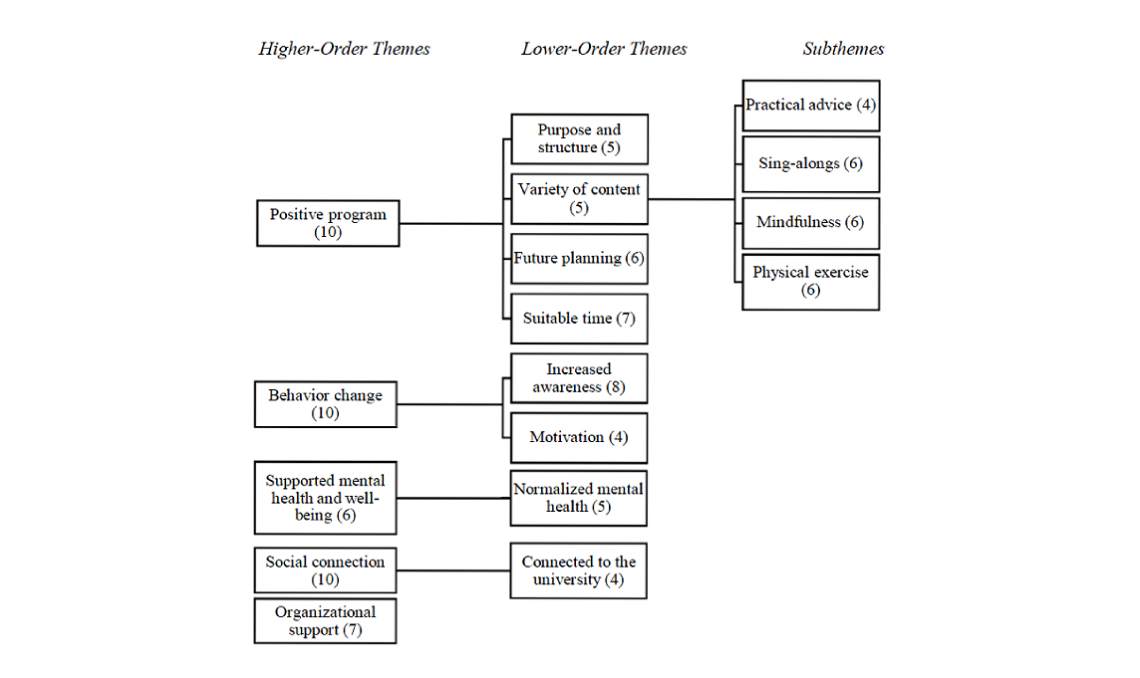
Themes from semistructured interviews with participants.

Participants in the semistructured interviews were aged, on average, 54 years (range 29-69 years), were predominantly professional staff (90%), and predominantly female (90%). Thematic analysis identified five higher-order themes: a positive program, facilitating behavior change, supporting mental health and well-being, providing social connection, and organizational support (Figure 1). Exploration of the higher-order themes, lower-order themes, and subthemes formed the basis of the findings.

#### A Positive Program

Four lower-order themes emerged from the higher-order theme of a positive program: (1) provided purpose and structure, (2) variety of relevant content, (3) future planning, and (4) suitable time. The VU Elevenses program was reported as a positive experience for participants during the COVID-19 pandemic. The program provided structure and purpose by supporting participants to have something to look forward to during extended periods of remote working and mandated isolation. The 11 AM time was suitable and the 15-minute duration was sufficient. Further, the program provided a variety of content, ranging from practical advice, sing-a-longs, mindfulness, and physical activities. This variety was reported as positive, as participants could opt in and out of the program depending on what suited their needs. Participants reported that the mix of content kept the program refreshing and engaging. Further, participants reported that they wanted the program to continue after the immediate response to the COVID-19 pandemic.

… It’s been mind-blowing. It’s been inspiring. It’s been educational. It’s taught me strategies. It’s given me physical, mental, and different perspectives to think about how to do things better during this lockdown COVID…it’s the best thing since sliced bread. It should continue. It should have happened sooner. So I think COVID has really pushed us to thinking, doing, thinking better in how we plan our days and to take care of ourselves

Participant 6

…bringing us back and reminding us to set some structure, have things to look forward to in the day, schedule in things that you want, and not just leave them to chance

Participant 3

I think there is so much value and benefit of what has happened with Elevenses that could continue on beyond, you know, what we are facing right now

Participant 4

#### Facilitating Behavior Change

Two lower-order themes emerged from the higher-order theme of facilitating behavior change: (1) increased awareness and (2) motivation. All participants described the VU Elevenses program as contributing to changing well-being behaviors during the COVID-19 pandemic. For example, some participants changed how they engaged with news and social media or incorporated breathing exercises into their daily lives. Participants felt the program created awareness and motivation for change, including regular prompts and reminders of strategies and skills they could implement, which facilitated implementing these changes.

…one of the strategies was about minimizing access to social media, particularly around the news. Not constantly looking at the news 'cause it is what it is. And so I think that was one that I found really useful to just think about where am I getting that information… finding one or two [trusted] sources… and that helped

Participant 2

…without really consciously thinking about making behavior change, I think that just having that kind of reminder was helpful

Participant 1

…overall I think the Elevenses, was very motivational. Every time I went into it, I left feeling very positive. So if there was a day where I felt unmotivated and I felt a bit like, “Ah, I can't be bothered,” when I'd go into the session, I'd leave feeling a bit more motivated

Participant 4

#### Supporting Mental Health and Well-being

One lower-order theme emerged from the higher-order theme of supporting mental health and well-being: normalizing mental health. Participants discussed how the program provided them with more confidence, insight, and understanding in managing their mental health and well-being during the COVID-19 pandemic. The online group program appeared to normalize any difficulties they were experiencing during the extended lockdown.

… my mental health has definitely adjusted and moved along the journey of the pandemic. So again, I think the program was very attuned to the situation and supporting mental health... I feel strongly that it did promote wellbeing and mental health in a very accessible way

Participant 3

… I think the breathing one, I sort of kept in my mind when I was feeling stressed, to do that. And then just the recognition that this has been identified as something that maybe other people might be going through as well helped

Participant 8

#### Providing Social Connection

One lower-order theme emerged from the higher-order theme of social connection: connected to the university. Participants reported that the program offered a space for staff to connect socially every day. Further, participants discussed feeling connected to the university and staff during the program, which seemed to provide a sense of comfort and familiarity.

… and it was also really great to see some of my colleagues that I haven’t been, physically been able to connect with, this made things really much better…

Participant 1

…made me feel a little bit more connected to the wider university because it made me feel more connected to what people were doing within the university

Participant 8

#### Organizational Support

Having access to the daily VU Elevenses program and feeling valued at work appeared to be beneficial for participants during the COVID-19 pandemic: “…that sense of connection to the organization and feeling like the organization actually values the staff has been the key thing for me.” [Participant 6]

### Longitudinal Changes in Depression, Anxiety, and Stress

A total of 106 participants completed the baseline questionnaire. At baseline, the average age of the 106 participants was 47.34 (SD 11.82) years and 92 (86.8%) identified as female. Overall, 79 (74.5%) participants were administrative or executive staff and 26 (24.5%) were teaching or research staff. The majority (70/106, 66.0%) were full-time staff. At baseline, 22.9%, 15.2%, and 25.2% of participants reported moderate-to-severe symptoms of depression, anxiety, and stress, respectively.

**Figure 2:**
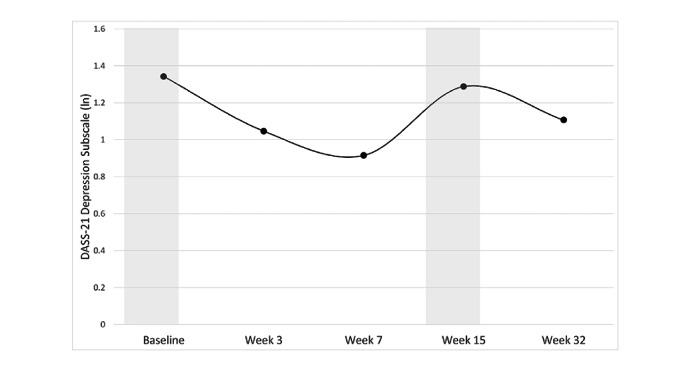
Change in depression scores during the program. The subscale indicates if outcomes were log-transformed (ln). Higher subscale numbers equate to greater symptoms. Time points shaded in grey indicate data collection periods during which participants were in strict lockdowns. DASS-21: Short Form Depression, Anxiety and Stress Scale.

**Figure 3:**
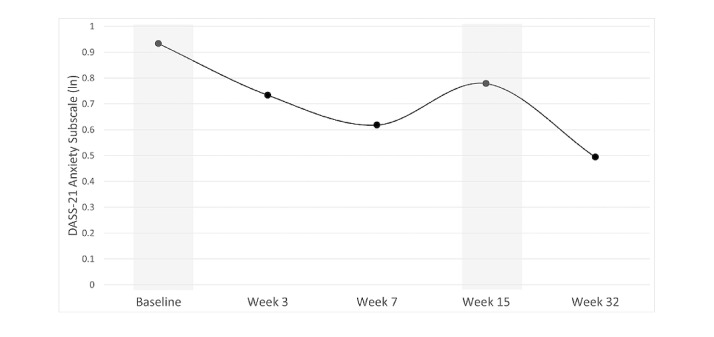
Change in anxiety scores during the program. The subscale indicates if outcomes were log-transformed (ln). Higher subscale numbers equate to greater symptoms. Time points shaded in grey indicate data collection periods during which participants were in strict lockdowns. DASS-21: Short Form Depression, Anxiety and Stress Scale.

**Figure 4:**
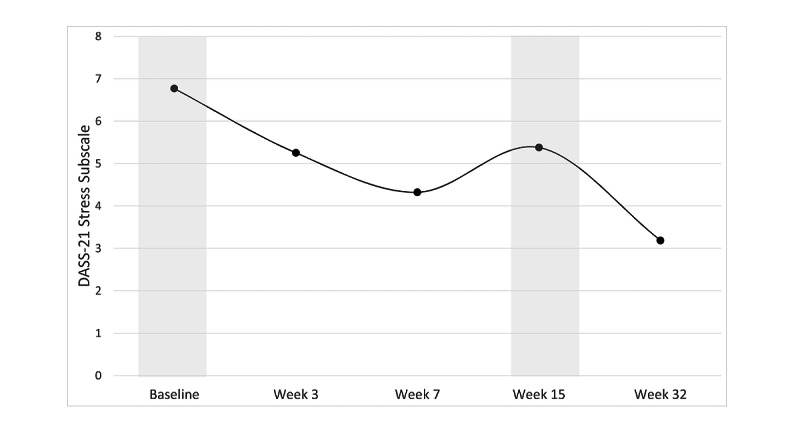
Change in stress scores during the program. DASS-21: Short Form Depression, Anxiety and Stress Scale.

Results from the multilevel linear growth models are shown in Table 2. The results showed that symptoms of depression, anxiety, and stress all followed a cubic trajectory decreasing from baseline to June 8, increasing from June 8 to July 31 (corresponding with a return to strict lockdown), and decreasing from July 31 to November 27 as lockdown restrictions were eased (Figures 2-4).

**Table 2:** Results from multilevel growth curve models for depression, anxiety, and stress.

Effects						Depression^a^			Anxiety^a^			Stress		
**Model A**														
	**Fixed effect: time**													
		B (95% CI)			–0.003 (–0.009 to 0.003)			–0.010 (–0.016 to –0.004)			–0.081 (–0.116 to 0.047)			
		*P* value			.39			.002			<.001			
	**Model fit**													
		–2LL		569.179			546.004			1408.421				
		Likelihood ratio (null model), *χ*^2^ (*df*=1)		0.752			9.501			20.581				
		*P* value		.39			.002			<.001				
**Model B**														
	**Fixed Effects**													
		**Time**												
			B (95% CI)		–0.017 (–0.039 to 0.006)			–0.019 (–0.041 to 0.004)			–0.154 (–0.278 to –0.031)			
			*P* value		.15			.02			.02			
		**Time^2^**												
			B (95% CI)		0.000 (–0.000 to 0.001)			0.000 (–0.000 to 0.001)			0.002 (–0.001 to 0.005)			
			*P* value		.21			.42			.23			
	**Model fit**													
		–2LL		567.637			545.385			1406.950				
		Likelihood ratio (Model A), *χ*^2^ (*df*=1)		1.542			0.619			1.471				
		*P* value		.21			.43			.23				
**Model C**														
	**Fixed effects**													
		**Time**												
			B (95% CI)		–0.137 (–0.194 to –0.080)			–0.090 (–0.149 to –0.031)			–0.678 (–1.000 to –0.356)			
			*P* value		<.001			.003			<.001			
		**Time^2^**												
			B (95% CI)		0.013 (0.007 to 0.018)			0.007 (0.002 to 0.013)			0.055 (0.024 to 0.085)			
			*P* value		<.001			.009			<.001			
		**Time^3^**												
			B (95% CI)		–0.000 (–0.000, to –0.000)			–0.000 (–0.000 to –0.000)			–0.001 (–0.001 to –0.000)			
			*P* value		<.001			.01			.001			
	**Model fit**													
		–2LL		548.578			538.925			1395.580				
		Likelihood ratio (Model A), *χ*^2^ (*df*=2)		20.601			7.079			12.841				
		*P* value		<.001			.03			.002				
		Likelihood ratio (Model B), *χ*^2^ (*df*=1)		19.059			6.460			11.370				
		*P* value		<.001			.006			<.001				

^a^Outcomes are log-transformed.

### Protective Factors Against Poor Mental Health

Results from the repeated-measures correlation (Multimedia Appendix 2) showed that COVID-19–related distress, self-compassion, and social connectedness had the strongest within-person correlations with symptoms of depression. The results from the mediation model for depression are presented in Table 3, showing a significant negative indirect effect of time through COVID-19–related distress on depression. There were no significant indirect effects through self-compassion or social connectedness. When controlling for mediators, there was no direct effect of time on symptoms of depression, indicating that changes in symptoms of depression were mediated by changes in COVID-19–related distress.

**Table 3:** Results from the multilevel mediation model on depression.

Effects	Path	B (95% CI)
Time through COVID-related distress	a1	–0.206 (–0.285 to –0.127)
COVID-related distress through depression	b1	6.228 (1.213 to 11.242)
Indirect effect	b1×a1	–1.299 (–2.558 to –0.040)
Time through self-compassion	a2	4.297 (2.228 to 6.366)
Self-compassion through depression	b2	–0.304 (–0.590 to –0.018)
Indirect effect	b2×a2	–1.284 (–2.579 to 0.011)
Time through social connectedness	a3	3.686 (0.755 to 6.617)
Social connectedness through depression	b3	0.001 (–0.200 to 0.202)
Indirect effect	b3×a3	–0.022 (–0.761 to 0.717)
Time through depression	c	1.144 (–0.450 to 2.737)

Repeated-measures correlation showed that COVID-19–related distress, self-compassion, and sleep quality had the strongest within-person correlations with symptoms of anxiety. Results from the mediation analysis for anxiety (Table 4) showed that there was also a significant negative indirect effect of time through COVID-19–related distress. There were no significant indirect effects through self-compassion or sleep quality. When controlling for mediators, there was no direct effect of time on symptoms of anxiety, indicating that changes in symptoms of anxiety were mediated by changes in COVID-19–related distress.

**Table 4:** Results from the multilevel mediation model on anxiety.

Effects	Path	B (95% CI)
Time through COVID-related distress	a1	–0.239 (–0.309 to –0.170)
COVID-related distress through anxiety	b1	7.659 (4.957 to 10.360)
Indirect effect	b1×a1	–1.828 (–2.585 to –1.071)
Time through self-compassion	a2	4.718 (2.972 to 6.464)
Self-compassion through anxiety	b2	–0.103 (–0.210 to 0.004)
Indirect effect	b2×a2	–0.480 (–1.005 to 0.044)
Time through sleep quality	a3	1.451 (0.741 to 2.162)
Sleep quality through anxiety	b3	–0.14 (–0.425 to 0.133)
Indirect effect	b3×a3	–0.215 (–0.675 to 0.246)
Time through anxiety	c	0.27 (–0.523 to 1.067)

Repeated-measures correlation showed that COVID-19–related distress, self-compassion, and diet quality had the strongest within-person correlations with stress. Results for the mediation analysis for stress (Table 5) showed that there was a significant indirect effect of time through self-compassion. There were no significant indirect effects through COVID-19–related distress or diet quality. When controlling for mediators, there was no direct effect of time, indicating that changes in stress were mediated by changes in self-compassion.

**Table 5:** Results from the multilevel mediation model on stress.

Effects	Path	B (95% CI)
Time through COVID-related distress	a1	–0.221 (–0.293 to –0.148)
COVID-related distress through stress	b1	5.189 (0.532 to 9.846)
Indirect effect	b1×a1	–1.156 (–2.335 to 0.024)
Time through self-compassion	a2	3.822 (1.702 to 5.942)
Self-compassion through stress	b2	–0.438 (–0.641 to –0.234)
Indirect effect	b2×a2	–1.662 (–2.745 to –0.580)
Time through diet quality	a3	0.831 (–0.075 to 1.738)
Diet quality through stress	b3	–0.341 (–0.907 to 0.225)
Indirect effect	b3×a3	–0.260 (–0.780 to 0.261)
Time through stress	c	–0.500 (–2.154 to 1.154)

## Discussion

### Principal Findings

This study examined the acceptability, outcomes, and potential mediators of daily, evidence-informed, online microinterventions to promote the mental health of staff of a tertiary education institution during the COVID-19 pandemic. Regarding program acceptability, results from weekly questionnaires showed that the vast majority of participants were satisfied with the program content, and believed that the content was helpful for their mental health and well-being. Additionally, qualitative interviews showed that participants had positive perceptions about the program’s structure and content, believed it supported their mental health and well-being, helped them implement positive behavioral change, and made them feel more socially connected and supported by the university. Results from the longitudinal analysis of mental health symptoms showed that participants had moderate-to-severe symptoms of depression, anxiety, and stress at baseline, and that symptoms declined over the first 7 weeks of the intervention. Additionally, although participants’ symptoms of depression, anxiety, and stress increased with the commencement of a second, stricter lockdown period, symptoms did not return to the levels reported at the beginning of the initial COVID-19 lockdown. This suggests that participation in the “VU Elevenses” program may have protected against worsening symptoms of depression, anxiety, and stress associated with mass lockdown, fears of an outbreak, and economic recession [33]. Additionally, results from the multilevel mediation analysis suggested that reductions in depression and anxiety were explained by reductions in COVID-19–related distress, and that participants who experienced greater increases in self-compassion over the study experienced greater reductions in symptoms of stress.

The moderate to severe symptoms of depression, anxiety, and stress reported by approximately 15%-25% of our participants at baseline are expected responses to the unknown and stressful circumstances of a health epidemic [2,34] and mandated self-isolation [35,36]. As stated by international mental health experts, a rise in these symptoms was predicted to occur during the COVID-19 pandemic, as well as an increase in the prevalence of diagnosed depression and anxiety disorders, self-harm behaviors, and suicide [34]. Indeed, the mental health impacts of the COVID-19 pandemic are apparent, with reported increases in the experience of psychological distress, symptoms of depression and anxiety, and incidence of mental disorders [37-39], and highlight the need for effective mental health supports during this time of crisis.

Results from this study indicate that a brief daily online intervention may be an acceptable way to provide mental health support during a time of crisis. Staff experienced the VU Elevenses program as a positive program during the COVID-19 pandemic, and the vast majority of staff agreed that they found the content helpful for their mental health and well-being. Similarly, high levels of satisfaction have been reported in multiple other online interventions during the COVID-19 pandemic [40,41], indicating that it may be viable to deliver mental health interventions online. Additionally, many participants agreed that they felt more connected with their peers and the university due to the VU Elevenses program. It is promising that our program, delivered during the period of mandated self-isolation, increased the sense of connection with colleagues. An adverse consequence of COVID-19 restrictions is the impact of social isolation and loneliness on mental health and well-being [35,36]. This is likely a consequence of the intervention being delivered in a group setting and allowing participants to connect with their peers daily, which can increase perceptions of social support during social isolation [42]. Additionally, by delivering the intervention in groups, the program may have normalized any difficulties staff may have been experiencing during the lockdown.

The longitudinal changes in mental health reported are similar to those reported in previous studies. Several studies reported worse mental health status at the beginning of the COVID-19 pandemic compared to prepandemic levels; however, mental health improved during the early months of the pandemic [43-45]. This may be because the greatest burden of lockdowns occurs early, and people may be able to adapt to their new situation over time [46]. Other studies have also reported a nonlinear trajectory in mental health, with mental health problems reemerging after the initial improvement [44]. This is likely to follow increases in case numbers and reintroductions of local lockdown measures, contributing to worsening mental health [44]. Although previous online mental health interventions reported short-term improvements in mental health during the COVID-19 pandemic [47-49], the results from this study indicate that these improvements may not be sustained long-term, especially if local cases began to rise and stricter restrictions are reintroduced. Therefore, rapid-response health promotion interventions need to be adapted to changing participant needs throughout a crisis period to be able to provide the most appropriate supports when they are required the most.

Results from the mediation analysis indicated that preintervention to postintervention changes in depression and anxiety were explained by changes in COVID-19–related distress. This suggests that the program may have provided participants with the strategies and skills to adapt their behaviors and regulate their emotions to reduce the distress associated with the pandemic. By contrast, changes in stress were explained by changes in self-compassion over time. The Elevenses intervention used several strategies to improve self-compassion, including self-compassion meditation, resetting expectations, and simple mindfulness strategies (Table 1). Self-compassion may promote a healthier and more adaptive style of thinking and decrease rumination [50], which may explain why it was associated with changes with stress over time.

In line with recommendations to share information on managing stress, depression, and anxiety using telehealth [2] or digital responses [34], our program delivered psychological and lifestyle strategies and was regularly accessed by a group of VU staff. The weekly evaluation demonstrated a high level of program acceptability and indicated that participants who engaged in the program perceived real benefits to their mental health and well-being. The uncontrolled intervention was offered each workday, and there was no requirement to attend each day, providing flexibility for individual circumstances.

### Strengths and Limitations

The content of our program was evidence-informed and was grounded in the six identified lifestyle intervention areas for well-being [11], including stress management, physical activity, nutrition, reducing substance use, sleep, and relationships. These influence coping, resilience, and mental health generally as well as in the context of stressful experiences [34]. The program’s content can be delivered in any workplace that has practitioners or clinicians on staff, can facilitate access to allied health professionals, or source and share appropriate existing online resources and clinical content. Despite the strength of having a flexible and responsive approach to content, aspects of the program that were based on general clinical content such as self-compassion, relaxation, and mindfulness practices could readily be reproduced. In addition, our findings demonstrate that it is possible to develop and deliver an acceptable program of online microinterventions in a timely manner, utilizing simple and accessible low-cost online meeting platforms, offered to an entire workplace at one time point. Until May 15, 2020, Australia’s federal government response was focused on managing health and financial needs rather than on the mental health implications of the COVID-19 crisis [2,51]. Our program was able to address this gap within our workplace, as it was developed and launched within 4 weeks from when staff were required to work remotely, and delivered wholly online to adhere to physical distancing and self-isolation requirements. This program allowed for the rapid collection of feedback from participants as well as timely sharing among the research team to adapt to anticipated changes in social regulations and restrictions. To the best of our knowledge, this is the first live, brief, weekday mental health promotion program in a workplace during a crisis.

Several limitations should be considered in the interpretation of these results. First, participants who opted into the program and the evaluation, including the semistructured interviews, may be subject to self-selection bias. Participants who value mental health promotion initiatives may be more likely to participate. Despite our intention for this program to be a universal mental health promotion and prevention intervention, only 18% of invited staff participated in the program, indicating modest reach into the target population [52]. Indeed, we do not have data to determine if our sample represents the larger staff body. The gender disparity in participation in the research component (females: 80% research opt-in vs 60% overall VU staff) may reflect the well-documented gender differences in help-seeking behaviors related to mental health, or reflect the attitudinal or societal barriers reported by men in accessing mental health support [53]. This should be considered for future program marketing and content development. As this program was developed as a rapid mental health promotion response, we could not develop an exhaustive internal promotion and engagement strategy. Inviting staff to participate via a general announcement email list can be problematic as many staff may not read these emails in detail. Follow-up responses to the opt-in research component were lower than expected and it is possible that the length of the survey may have contributed to this low response rate. As an uncontrolled intervention without a comparison group, it is possible that the changes in psychological symptoms reflect natural adaptation to new routines and life circumstances over time. This is reflected in the results of the mediation models, which showed that changes in COVID-19–related distress explained changes in depression and anxiety from baseline to the study endpoint.

### Conclusions

This study demonstrates the acceptability of a rapidly developed program of microinterventions, delivered in a timely manner utilizing a simple and accessible low-cost online meeting platform during a time of crisis when mental health is highly likely to deteriorate. Participation in the program may have protected against worsening symptoms of depression, anxiety, and stress associated with extended COVID-19 lockdown periods, and improved symptoms of stress by increasing self-compassion.

## Appendix

Multimedia Appendix 1 Participant semistructured interviews schedule.

Multimedia Appendix 2 Repeated-measures correlation between factors associated with mental health and symptoms of depression, anxiety, and stress.

## Abbreviations

DASS-21: Short Form Depression, Anxiety and Stress Scale
MSPSS: Multidimensional Scale of Perceived Social Support
SCS: Social Connectedness and the Social Assurance Scales
SCS-SF: Self-Compassion Short Form
VU: Victoria University
