# Supplementary material for: A Brief, Daily, Online Mental Health and Well-being Intervention for University Staff During the COVID-19 Pandemic: Program Description and Outcomes Using a Mixed Methods Design
Source: JMIR Form Res. 2022 Feb 25;6(2):e35776. doi: 10.2196/35776 (PMC8887557; doi:10.2196/35776)
Supplement: Multimedia Appendix 1 [file formative_v6i2e35776_app1.docx]

**Appendix A**

**Participant semi-structured schedule**

1. What is your overall perception of the VU Elevenses program? What has the experience been like for you?
2. How did you hear about the VU Elevenses program? What influenced your decision to participate in the program?
3. With the objectives of promoting mental health and wellbeing by providing helpful strategies and increasing connection for VU staff during the COVID-19 pandemic, what are your thoughts on whether or not these objectives have been met?
4. The program is unique in offering daily (weekday), brief, online sessions. Some participants have attended the sessions regularly and others less so. Can you tell us about your level of engagement with the program? Did it change over time? What influenced your attendance? [Prompt: average number of sessions attended, daily 11am suitable/not suitable, content relevant/irrelevant, online format, consistency/routine]
5. Have you recommended the program to your colleagues? Why/why not?
   1. Why do you think some of your colleagues chose not to participate?
6. The VU Elevenses program was informed by behaviour change techniques with the aim of assisting participants to increase healthy behaviours across physical activity, nutrition, sleep, relationships, alcohol and stress management. How useful and beneficial was this type of content for you during the COVID-19 pandemic on a general level?
7. What sessions or strategies delivered in the program were the most useful and beneficial for your mental wellbeing? [Prompt for specific techniques (eg mindfulness, relaxation, sleep), sessions (eg physical activity, sing-alongs, wellness planning, social isolation), connection with colleagues]
   1. In what way did these sessions provide you with skills, strategies or techniques you could try out to promote your mental health and wellbeing? [Prompt: did you try any of the suggestions? Did you attempt anything new?]
   2. Changing behaviours or trying new things can be a challenge. What are some of things you noticed that influenced your motivation or ability to implement some of these strategies in your everyday life?
   3. In what way did the content impact on your mental health and wellbeing? Did it help to reduce any COVID-19 related stress or worries you may have been experiencing? [Prompt: did your feelings change in any way after trying a new strategy?]
   4. In what way did trying a new strategy or skill help to more confident about managing your mental wellbeing? [Prompt: did it change your views on how you are coping?]
   5. Do you intend to keep trying to engage with the content, strategies and techniques introduced through the VU Elevenses program? How difficult do you think it will be to maintain the changes you have made? Can you think of anything that might help you to maintain the changes? [Prompt: preparation, routine, involving others, setting new goals, monitoring progress]
   6. Did you share any of the content, strategies, or techniques with family or friends? What was this experience like? How did they respond?
8. The VU Elevenses also included additional online resources on our intranet page as well as recordings of previous sessions.
   1. Did you view or use any of the additional resources or links? Which ones?
   2. Did you view any of the recording sessions? Sessions you missed? Or sessions you wanted to view again? How often? Which ones?
9. How well did the VU Elevenses program overall fit with your idea of what looking after your mental health and wellbeing during COVID-19 would be like? [Prompt: what is your reaction to some of the content; eg physical activity, eating well, stress management/mindfulness, sing-alongs/quizzes?]
10. How useful were the physical activity and exercise snacking sessions? What were some of the factors that influenced your participation in these sessions specifically?
    1. Have these regular sessions changed your activity levels during COVID-19? [Prompt for levels of PA, have they used techniques (eg stretching, cardio, resistance exercises) outside of VU Elevenses sessions?]
11. What other resources have your used for your mental health and wellbeing during this time? [Prompt: informal supports from family and friends, formal help-seeking (GP, allied health), using VU employee assistance program, online programs or apps]
12. What suggestions would you make for the VU Elevenses program to be more helpful, satisfying or engaging to VU staff?
    1. Feedback on specific presenters?
    2. Online meeting format?
    3. Session content?
    4. Session duration?
    5. Are there any aspects of the program that you hope could continue post the immediate response to the COVID-19 pandemic?
13. If you had the chance to design a mental health and wellbeing program for VU staff during the COVID-19 pandemic, what approach would you have taken?
    1. How does the VU Elevenses match your perceptions or expectations of what a mental health promotion program should be like?
14. Is there any other feedback about the VU Elevenses program that you would like to give us?
