# Supplementary material for: A Brief, Daily, Online Mental Health and Well-being Intervention for University Staff During the COVID-19 Pandemic: Program Description and Outcomes Using a Mixed Methods Design
Source: JMIR Form Res. 2022 Feb 25;6(2):e35776. doi: 10.2196/35776 (PMC8887557; doi:10.2196/35776)
Supplement: Multimedia Appendix 2 [file formative_v6i2e35776_app2.docx]

**Appendix B**

Repeated measures correlation between factors associated with mental health and symptoms of depression, anxiety and stress.

|  | Depression | Anxiety | Stress |
| --- | --- | --- | --- |
|  |  |  |  |
| COVID distress | 0.46** | 0.76*** | 0.53** |
| Self-compassion | -0.58*** | -0.58*** | -0.82*** |
| Social Support | -0.03 | -0.10 | 0.11 |
| Social Connectedness | -0.42* | -0.42* | -0.49** |
| MVPA | -0.32 | -0.07 | -0.29 |
| Active Behavioural Coping | -0.09 | -0.18 | 0.01 |
| Active Cognitive Coping | -0.03 | -0.07 | -0.11 |
| Avoidance Coping | -0.06 | -0.01 | 0.18 |
| Diet | -0.39* | -0.40* | -0.54** |
| Sleep Quality | -0.34 | -0.45** | -0.52** |
| Alcohol Use | -0.00 | -0.16 | -0.12 |

* *P*<.05, ** *P*<.01, *** *P*<.001
